# The default mode network and social understanding of others: what do brain connectivity studies tell us

**DOI:** 10.3389/fnhum.2014.00074

**Published:** 2014-02-24

**Authors:** Wanqing Li, Xiaoqin Mai, Chao Liu

**Affiliations:** ^1^State Key Laboratory of Cognitive Neuroscience and Learning and IDG/McGovern Institute for Brain Research, Beijing Normal UniversityBeijing, China; ^2^Center for Collaboration and Innovation in Brain and Learning Sciences, Beijing Normal UniversityBeijing, China; ^3^Department of Psychology, Renmin University of ChinaBeijing, China

**Keywords:** default mode network, social cognition, brain connectivity, morality, theory of mind, empathy

## Abstract

The Default Mode Network (DMN) has been found to be involved in various domains of cognitive and social processing. The present article will review brain connectivity results related to the DMN in the fields of social understanding of others: emotion perception, empathy, theory of mind, and morality. Most of the reviewed studies focused on healthy subjects with no neurological and psychiatric disease, but some studies on patients with autism and psychopathy will also be discussed. Common results show that the medial prefrontal cortex (MPFC) plays a key role in the social understanding of others, and the subregions of the MPFC contribute differently to this function according to their roles in different subsystems of the DMN. At the bottom, the ventral MPFC in the medial temporal lobe (MTL) subsystem and its connections with emotion regions are mainly associated with emotion engagement during social interactions. Above, the anterior MPFC (aMPFC) in the cortical midline structures (CMS) and its connections with posterior and anterior cingulate cortex contribute mostly to making self-other distinctions. At the top, the dorsal MPFC (dMPFC) in the dMPFC subsystem and its connection with the temporo-parietal junction (TPJ) are primarily related to the understanding of other's mental states. As behaviors become more complex, the related regions in frontal cortex are located higher. This reflects the transfer of information processing from automatic to cognitive processes with the increase of the complexity of social interaction. Besides the MPFC and TPJ, the connectivities of posterior cingulate cortex (PCC) also show some changes during tasks from the four social fields. These results indicate that the DMN is indispensable in the social understanding of others.

## Introduction

### The default mode network and social understanding of others

Human beings are social animals that have a tendency to interpret stimuli according to their possible social relevance, and spend a huge amount of time assessing one's own and other's social relationships and positions by engaging in activities such as thinking about oneself and others and exchanging those thoughts during the whole of life (Schilbach et al., 2008). Dunbar and coleagues suggested a “social brain hypothesis,” which deemed that the large brains observed in primates reflected the computational demands of the complex social systems that characterized the order of their members (Dunbar, 1993).

In the past two decades, the social brain of human has been intensively studied in several different domains: (1) understanding others, (2) understanding oneself, (3) controlling oneself, and (4) the processes that occur at the interface of self and others (Lieberman, 2007). However, in the strictest sense, social cognition is about understanding of other people, including their emotional, mental, psychological status, and behaviors (Lieberman, 2007). Increasing studies have shown that regions of the default mode network (DMN) largely activate in tasks requiring participants to understand and interact with others, such as perceiving and interpreting other's emotion status, showing empathy to other people, inferring other's belief and intention, and performing moral judgments on other's behavior (Schilbach et al., 2008; Laird et al., 2011). Besides overlaps with the DMN, the large scale brain networks for social domains also contain several regions outside the DMN, since these social behaviors usually comprise extensive cognitive processes such as obtaining, retrieving, and processing information about the lives, relationships, and mental states of others (Mars et al., 2012).

In the present article we will review results related to the DMN in the field of social understanding of others using brain connectivity methods. Several important fields of social behavior, emotion perception, empathy, theory of mind (ToM, or mentalizing), and morality, will be summarized for both healthy subjects and patients with autism, psychopathy and schizophrenia (see Table 1). The existing results were organized through two aspects. The first one is how the regions within the DMN interact with each other when people perform those social tasks, and the second one is how the DMN interacts with other distributed brain systems that contribute to the process of social cognition of others. Possible future directions will be discussed at the end.

**Table 1:** **Brain connectivity studies on the social understanding of others**.

**Study**	**Paradigm**	**Method**	**Connectivity within the DMN**	**Connectivity between DMN and other regions**	**Number of subjects**	**Results**
**EMOTION PERCEPTION**						
Etkin et al., 2006	Emotional stroop task	EC, PPI, voxel-wise DCM		rostral ACC-amygdala	19	↑EC from rACC to amygdala during high conflict, the strength predicted successful conflict resolution
Passamonti et al., 2008	Emotional faces gender decision	EC, PPI, voxel-wise DCM		vACC-amygdala	21	EC from vACC to amygdala negatively correlated to reward-drive score
Das et al., 2005	Fear perception	FC, PPI, seed-based		vACC, and dACC with thalamus–sensory cortex pathway, and thalamus–amygdala pathway	28	Positive modulation from dACC and negative relationship from vACC on thalamus–sensory cortex pathway; both dorsal and vACC had inverse interaction with thalamus–amygdala pathway
Cremers et al., 2010	Emotional faces gender decision	FC, PPI, voxel-wise		dMPFC-amygdala; ACC-amygdala	60	Neuroticism scores positively correlated with FC of dMPFC-right amygdala for angry and fearful faces, and negatively correlated with FC of ACC-left amygdala for angry, fearful, and sad faces
Satterthwaite et al., 2011	Emotion identification	FC, PPI, voxel-wise		Medial OFC-amygdala, MPFC-amygdala	39	Positive FC of medial OFC-amygdala, and negative FC of MPFC-amygdala during task
Kleinhans et al., 2008	Face identification	FC, seed-based		PCC-FFA	47 (24 autistic)	↓FC in ASD group
Rudie et al., 2012	View emotional face expressions	FC, seed-based		vMPFC-rIFGpo	47 (23 autistic)	↓negative FC in ASD group
**EMPATHY**						
Decety et al., 2008	View pain scenarios	EC, PPI, voxel-wise	Medial OFC-right TPJ, ParaCC-right TPJ	Medial OFC-anterior IPS, precentral sulcus, and anterior MCC; ParaCC-anterior IPS, and precentral sulcus	17	↑EC during condition of pain which was caused intentionally compared to pain which occurred accidentally
Otti et al., 2010	View pain scenarios	FC, ICA	Within anterior DMN		19	↓FC from “No Pain” to “Pain,” and the strength positively correlated with the subjective post-scan pain
Zaki et al., 2007	Experience self pain, and view other pain	FC, PPI, voxel-wise		MPFC, PCC-AI, dACC	19	↑FC from self pain task to other pain task
Cheng et al., 2007	View pain scenarios	FC, PPI, seed-based		MPFC-insula	28 (14 experts)	↑negative FC in the experts compared to control
Meyer et al., 2013	View social pain scenarios	FC, PPI, voxel-wise		MPFC-AI, MPFC-dACC	16	↑FC for the friend's exclusion
Gu et al., 2010	View pain scenarios	FC, PPI, seed-based		Superior MPFC-frontoinsula	18	↓FC under the context of painful stimuli
Cox et al., 2012	Self-report of empathy	FC, seed-based		Perigenual ACC-left amygdala	38	Dominance of affective empathy was related to stronger positive FC, dominance of cognitive empathy was related to stronger negative FC
Akitsuki and Decety, 2009	View pain scenarios	FC, PPI, voxel-wise		Medial OFC-amygdala, precuneus-amygdala	26	↑FC of medial OFC-left amygdala, precuneus-left amygdala during painful situations caused intentionally
**THEORY OF MIND**						
Atique et al., 2011	Emotion, intention ToM	FC, seed-based	vMPFC-anterior TPJ		24	↑FC of vMPFC-anterior TPJ during emotion mentalizing
Burnett and Blakemore, 2009	Imagine basic and social emotional experience	FC, PPI, seed-based, voxel-wise	Anterior rostral MPFC-pSTS/TPJ		28 (10 adults)	↑FC during social emotion both in adolescents and adults, and ↑FC in adolescents compared to adults during social emotion
Mason et al., 2008	Read passages	FC, seed-based	MPFC-TPJ	Left hemisphere language network-ToM network	36 (10 autistic)	↓FC between left MPFC and right TPJ, as well as left hemisphere language network and ToM network, during intentional inference condition in the autistic group
Baumgartner et al., 2012	Punish people for violating social norms	FC, PPI, Seed-based	dMPFC-left TPJ		16	Negative correlation between FC of dMPFC-left TPJ and third-party punishment of defecting in group members
Das et al., 2012	Infer states of two moving triangles	FC, ICA		Posterior DMN-lateral fronto-temporal networks and insula	45 (23 schizophrenic)	↓FC in schizophrenic
Herve et al., 2012	Comprehend affective speech	FC, seed-based	MPFC-TPJ	“Medial” network –“Language” network, amygdala	51	Interaction between language (inferior frontal, and temporal areas), ToM (MPFC, TPJ), and emotion processing network observed during emotional speech comprehension
Lombardo et al., 2010	ToM judgments about self or a familiar non-close other	FC, seed-based			33	vMPFC, PCC/precuneus, and TPJ exhibited same FC patterns during mentalizing of both self and other
**MORALITY**						
Pujol et al., 2012	Resting state, moral dilemma, stroop task	FC, seed-based	MPFC-PCC		44 (22 psychopaths)	↓FC during resting state in psychopathic group
Craig et al., 2009		DTI		OFC-amygdala	27 (18 psychopaths)	↓FA of the uncinate fasciculus in psychopaths
Marsh et al., 2011	Moral judgment implicit association	FC, seed-based		rACC/OFC-amygdala	28 (14 psychopaths)	↓FC during task performance in psychopaths
Decety et al., 2012a	View moral scenarios	FC, PPI, seed-based	vMPFC-TPJ	vMPFC-amygdala	126	↑FC of vMPFC-amygdala with age when viewing intentional harm, ↑FC of vMPFC-pSTS/TPJ while viewing moral actions in adults compared to adolescents
Verdejo-Garcia et al., 2012	Resting state, moral dilemma	FC, seed-based cross-correlation analysis		ACC-thalami	24 (cocaine users)	↓FC during resting state in cocaine-dependent subjects
Shannon et al., 2011		FC, IDEA		DMN-PMdr	202 (107 offenders)	FC positively correlated with impulsivity score in juvenile offenders, while negatively correlated with age in typical developing individuals

PPI, psychophysiologic interaction analyses; DCM, dynamic causal modeling; ICA, independent component analysis; IDEA, iterative data-driven evolutionary algorithm; FC, functional connectivity; EC, effective connectivity; ASD, autism spectrum disorder; DTI, diffusion tensor imaging; MPFC, medial prefrontal cortex; vMPFC, ventral medial prefrontal cortex; dMPFC, dorsal medial prefrontal cortex; PCC, posterior cingulate cortex; ACC, anterior cingulate cortex; rACC, rostral anterior cingulate cortex; vACC, ventral anterior cingulate cortex; dACC, dorsal anterior cingulate cortex; OFC, orbital frontal cortex; TPJ, temporo-parietal junction area; IPS, intraparietal sulcus; MCC, midcingulate cortex; ParaCC, paracingulate cortex; AI, anterior insula; pSTS, posterior superior temporal sulcus; IFG, inferior frontal gyrus; MTG, middle temporal gyrus; PMdr, dorsolateral premotor cortex; AG, angular gyrus; SFG, superior frontal gyrus; FFA, fusiform face area; rIFGpo, the right pars opercularis of the inferior frontal gyrus; PHC, parahippocampal cortex.

### The default mode network

**Figure 1:**
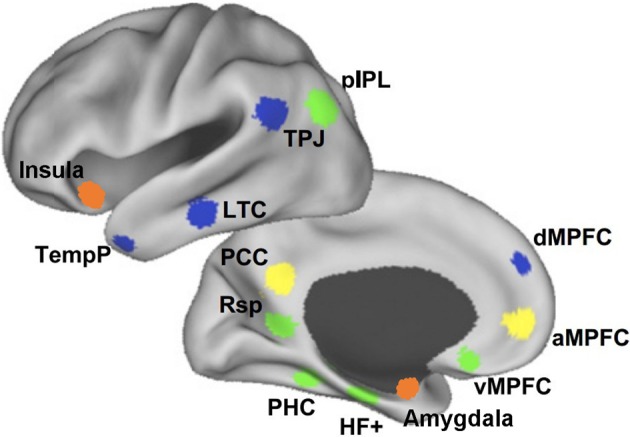
**The medial prefrontal cortex (MPFC) plays a key role in the social understanding of others**. The subregions of MPFC belong to different subsystems of DMN. At the bottom, the ventral MPFC is in the medial temporal lobe subsystem (green) and its connections with emotion regions are mainly associated with emotion engagement during social interactions. Above, the anterior MPFC is in the cortical midline structures (yellow) and its connections with posterior and anterior cingulate cortex contribute mostly to making self-other distinctions. At the top, the dorsal MPFC (dMPFC) is in the dMPFC subsystem (blue) and its connection with the temporo-parietal junction (TPJ) are primarily related to understanding others' mental states (Andrews-Hanna et al., 2010b). The orange clusters show the anterior insula and amygdala, which contribute greatly in the social understanding of others.

The DMN is an anatomically defined brain system that preferentially activates when individuals are not focused on the external environment (Buckner et al., 2008). Core areas of the DMN include the medial posterior cortex [specifically the posterior cingulate cortex (PCC) and parts of the precuneus], medial prefrontal cortex (MPFC), as well as bilateral inferior parietal lobule (IPL) expanding to posterior temporal areas around the temporo-parietal junction (TPJ). Apart from these core areas, hippocampus and adjacent regions in the medial temporal lobe (MTL) and lateral temporal cortex (LTC) extending toward the temporal pole (TP) are also often reported as part of the DMN (Shulman et al., 1997; Buckner et al., 2008; Andrews-Hanna et al., 2010b) (see Figure 1).

The DMN was originally identified in a meta-analysis mapping brain areas that showed increased activity during passive tasks compared to active tasks in block-design positron emission tomography (PET) studies (Shulman et al., 1997). Three kinds of activity patterns within the DMN have been found since then. The first one is consistently decreased activity when subjects engage in goal-directed tasks as compared to control states (Gusnard and Raichle, 2001; Greicius et al., 2003); the second one is the high intrinsic activity during resting states with the eyes closed or visually fixating without engagement in any specific task (Raichle et al., 2001; Greicius et al., 2003; Beckmann et al., 2005); and the last one is the striking overlap between the DMN and regions activated in social cognitive tasks (Schilbach et al., 2008; Eickhoff et al., 2009).

So far, evidence have been found that brain regions within the DMN contribute to specialized functions organized into subsystems that converge on hubs. Buckner et al. (2008) pointed out that the DMN consisted of at least two interacting subsystems: the MTL subsystem containing both the hippocampal formation (HF) and parahippocampal cortex (PHC); and the core MPFC subsystem including the posterior cingulate/retrosplenial cortex (PCC/Rsp), ventral MPFC (vMPFC), and IPL. They proposed that the MTL subsystem was associated with mnemonic processes and activated during successful retrieval of old information, and the MPFC subsystem was activated in tasks requiring participants to engage in self-relevant mental simulations. Using memories and associations from past experiences as its building blocks, the DMN participated in constructing self-relevant mental simulations that were exploited by a wide range of cognitive functions including remembering the past, thinking about the future, and conceiving the current viewpoint of others. Andrews-Hanna and colleagues further suggested that the DMN consisted of two subsystems that interacted with a common core system (see Figure 1): one was the dorsal MPFC (dMPFC) subsystem comprising the dMPFC, TPJ, LTC, and TP; and the other was the MTL subsystem comprising the HF, PHC, Rsp, vMPFC, and posterior IPL. The dMPFC subsystem was selectively activated when participants considered one's own and others' present mental states, whereas the MTL subsystem showed preferential activity when participants simulated the future using mnemonic imagery-based processes. Both of these two subsystems were strongly correlated with a midline common core system consisting of the anterior MPFC (aMPFC) and PCC, which is usually activated when people make self-relevant affective decisions. The midline core system interacted with the MTL subsystem and the dMPFC subsystem to facilitate the construction of mental models of personally significant events (Andrews-Hanna et al., 2010b).

### Measuring brain connectivity in the DMN

An increasing number of researchers are interested in the brain connectivity among the DMN regions and have applied several newly developed approaches and methodologies to DMN studies. In the functional connectivity (FC) approach, researchers compute the statistical interrelation of neurophysiological time series representing temporal changes in different brain regions, and examine the stimulus-dependent and -independent synchronizations and interactions between these regions (Friston, 2005; Menon, 2011). In the effective connectivity (EC) approach, data can be obtained by dynamic causal modeling (DCM), which estimates and judges the negative or positive impacts of one region on another and how such impacts are affected by experimental context (Aertsen et al., 1989; Friston, 2002). Granger causality and other similar methods, unlike the bidirectional functional connectivity, which is a model-free concept, computes the unidirectional EC and emphasizes asymmetric causal interactions between neural systems. Granger causality estimates forward (bottom-up) vs. backward (top-down) connectivity between diverse regions. Nevertheless, it has been criticized for the lack of a biologically-based generative model and likelihood of obtaining pseudo estimated “causality” that is in fact induced by systematic differences across brain areas in hemodynamic lag (Friston, 2009; Smith et al., 2011). Functional and effective connectivity can be studied through both linear techniques (correlation coefficient, coherence) and non-linear techniques (phase synchronization, generalized synchronization) (Stam and van Straaten, 2012). It is worth noting that, negative correlations in brain connectivity analysis, sometimes referred to as anti-correlations, must be cautiously interpreted since they are usually present only after regressing whole-brain signals. This raises a point of controversy: whole-brain normalization leads to a bell-shaped correlation value distribution centered on zero, thereby guaranteeing negative correlations even if such correlations were not initially present in the data (Murphy et al., 2009; van Dijk et al., 2010). A brain network can also be defined on the basis of structural connectivity through Magnetic Resonance Imaging (MRI) morphology and Diffusion Tensor Imaging (DTI) tractography *in vivo* or tracer studies on postmortem tissue. Structural connectivity denotes a network of anatomical links and places constraints on which functional and effective interactions occur in the network (Bullmore and Sporns, 2009; Bressler and Menon, 2010; Menon, 2011).

## Brain connectivity studies on DMN and social understanding of others

### Emotion perception

Emotion plays a crucial role in human social cognition. Perceiving and interpreting other people's emotion status is one of the most important steps during social interaction. Traditional studies on the neural mechanism of emotion adopted a *locationist approach*, which asserted that each basic emotion faculty has its own specialized neural circuitry that is architecturally distinct, inborn, and shared with other animals (Panksepp, 2004). Early neuroimaging results were indeed congruent with this assumption, for example amygdala for fear (Adolphs et al., 1995), insula for disgust (Wicker et al., 2003), orbitofrontal cortex (OFC) for anger (Murphy et al., 2003), and subgenual anterior cingulate cortex (ACC) for sadness (Murphy et al., 2003). However, several recent meta-analyses and reviews favored the psychological *constructionist approach*, which suggested that a set of interacting brain regions involved in the basic psychological operations of both emotional and non-emotional processing were activated during emotion experience and perception (Lindquist and Barrett, 2012; Lindquist et al., 2012). Yet co-activation of different brain regions does not necessarily mean connectivity between them, so the evidence for the constructionist approach is inconclusive and brain connectivity results would be critical for examining this approach.

Most emotion perception studies using brain connectivity methods revealed changes between the DMN and other brain systems, especially between the prefrontal cortex and amygdala. In a gender discrimination task of angry and neutral faces, Passamonti et al. (2008) confirmed that the interaction between the ventral ACC and amygdala was influenced by the drive to obtain reward, with reduced negative connectivity in high reward-drive participants. The direction of this effect was limited to connection from the ventral ACC to the amygdala but not vice versa. Moreover, in another study, the rostral ACC was negatively coupled with the amygdala in high vs. low conflict resolution trials of a classic emotion Stroop task with fearful and happy faces, and the strength of the connectivity predicted successful conflict resolution (Etkin et al., 2006). Studies also found that the connectivity between different subregions of the MPFC and amygdala may make diverse effects on emotion function. For example, when people did a fear perception task, there was a dorsal-ventral division in ACC modulation of the thalamus-sensory cortex pathway, with a positive modulation of this pathway from dorsal ACC and a negative one from the ventral ACC (Das et al., 2005). In addition, Satterthwaite et al. (2011) demonstrated that the amygdala responded preferentially to threatening (fearful or angry) faces and had increased connectivity during threat trials with the OFC. Moreover, a study also showed that the neuroticism scores of subjects were negatively related to the left amygdala-ACC connectivity, but positively associated with the right amygdala-dorsomedial prefrontal connectivity, when processing negative emotional facial expressions (angry and fearful compared to neutral faces) (Cremers et al., 2010).

Besides the prefrontal cortex and amygdala, functional connectivity changes between other regions were also found in autism patients. For example, in a facial expression identification task, the healthy control group had significantly increased connectivity between the fusiform face area and PCC compared to autism patients (Kleinhans et al., 2008). In another study requiring subjects to passively view emotional facial expressions, typically developing children showed an anticorrelation between the right pars opercularis of the inferior frontal gyrus (rIFGpo) and the DMN, whereas autistic children showed a similar anticorrelated relationship between the rIFGpo and the posterior portion of the DMN, but not the anterior portion of the DMN (vMPFC) (Rudie et al., 2012).

General speaking, the FC in emotion perception studies concentrated on the relation between the vMPFC (including parts of ACC), and other emotion-related areas, mainly the amygdala and insula. The DMN has been theorized to make sensory inputs meaningful as “situated conceptualizations” for distinct emotions, since it reconstitutes past experiences for use in the present (Lindquist and Barrett, 2012; Lindquist et al., 2012). The vMPFC, as part of the DMN, is believed to receive reinforcement expectancy information from emotion learning systems that process the reinforcement provided by specific reinforcers of emotional expressions (Blair, 2007). Thus, the above results from functional connectivity in emotion perception may demonstrate that the vMPFC is indeed associated with successful regulation of human's emotional perception and responses.

### Empathy

Empathy can be defined as the process to generate an isomorphic affective state in the self to understand another individual's emotional state or condition while realizing that it is the other who causes this affective state (Decety and Svetlova, 2012; Engen and Singer, 2012). Neuroimaging of empathy is usually acquired by scanning people's brain when they fall into empathic states with various emotions such as disgust, reward, joy, and, particularly, pain (Jabbi et al., 2008; Singer et al., 2009; Bernhardt and Singer, 2012). Researchers proposed that at least three neural systems play vital roles in empathy: the mirror neuron system, the affective empathy system located in the anterior insula (AI) and midcingulate cortex (MCC), and the cognitive empathy system of theory of mind that almost overlaps with the DMN network. The affective empathy system and the cognitive empathy system are linked through the vMPFC (Walter, 2012).

Only a few empathy studies adopted brain connectivity methods to investigate the FC within the DMN, most of which were studying pain. For instance, although temporal correlation analysis demonstrated that the anterior DMN (aDMN) was deactivated in both the “Pain” and “No Pain” conditions compared to the resting-state, the decrease of connectivity was significantly stronger in the “No Pain” than “Pain” condition. In addition, independent component analysis (ICA) demonstrated that higher integration of the left medial OFC into the aDMN was associated with higher post-scan pain ratings (Otti et al., 2010).

Most of empathy studies focused on the connection between the DMN (e.g., MPFC) and other regions, especially the insula. When participants watched short videos of other people suffering painful injuries, the brain area of dMPFC and PCC showed greater connectivity with the dorsal ACC and AI than when participants received noxious thermal stimulation (Zaki et al., 2007). In another study, subjects were asked to view color photographs describing human body parts in painful or non-painful situations and then judge whether the person was suffering from pain or not. Results revealed that the frontoinsular cortex showed decreased FC with the superior MPFC in response to the painful compared to non-painful stimuli (Gu et al., 2010). Moreover, observing a friend experiencing social exclusion would trigger greater intensity of FC between the MPFC and both the dorsal ACC and bilateral insula than observing a stranger doing so (Meyer et al., 2013). Furthermore, Cheng et al. (2007) proposed that medical experts who applied painful procedures in their practice could regulate the unpleasant feelings generated by perceiving others in pain through modulating attentional demands. In accord with this hypothesis, experts showed negative FC between the MPFC and AI, whereas the controls showed no significant correlation with the MPFC.

As to the relationship between the amygdala and MPFC in empathy, studies found that the FC pattern between the amygdala and other brain areas was modulated by social context. For instance, the medial OFC and precuneus showed stronger covariation with the left amygdala when the visual stimulus was one person in a painful situation caused by another individual than when the situation was caused by accident (Akitsuki and Decety, 2009). Cox et al. (2012) argued that relative empathic ability (REA), the difference between affective empathy and cognitive empathy, is a useful index for empathy ability. Their results showed that the dominance of affective empathy was associated with stronger FC among social-emotional regions (ventral anterior insula, OFC, amygdala, perigenual ACC), whereas the dominance of cognitive empathy was related to stronger FC among areas implicated in social-cognitive regions (brainstem, STS, ventral anterior insula).

The FC differences found in empathy studies may reflect similar mechanisms as emotion perception, which involve the vMPFC's connection with the amygdala and insula (Akitsuki and Decety, 2009; Otti et al., 2010). Empathy has a deep evolutionary foundation stemming from the phylogenetically ancient practice of parental care, which assists the propagation of genetic legacy to future generations. The motivational systems originally developed to care for one's offspring have gradually been used to facilitate positive relationships between unrelated group members. Ultimately, empathy became a useful means of forming and maintaining strong social bonds between unrelated individuals (Decety et al., 2012b). By enabling human beings to feel the suffering of others, empathy can promote affective interactions and contribute to prosocial behaviors toward other conspecifics, depending on relevant social contexts and social relationships (Decety and Porges, 2011). Thus, it is very important for humans to identify the real protagonist of emotion—the one who causes this affective state. It follows that empathy, to a great extent, is based on emotion perception. Consistent with this line of thought, the region in the frontal cortex that is strongly implicated in both empathy and emotion perception is the aMPFC (Cheng et al., 2007; Otti et al., 2010; Cox et al., 2012; Meyer et al., 2013), which takes charge of the self-other distinction. There are also some other areas connected with the dMPFC (Zaki et al., 2007; Gu et al., 2010), which contribute to the recognition of other humans' mental states.

### Theory of mind

Theory of mind refers to the ability to explain, predict, and interpret another person's behavior by attributing affective and cognitive mental states such as desires, beliefs, intentions and emotions to other people (Amodio and Frith, 2006; Abu-Akel and Shamay-Tsoory, 2011; Krause et al., 2012). The machinery of ToM involves at least three basic processes: representing cognitive and affective mental states, attributing these mental states to others, and finally applying (or deploying) these mental states to correctly comprehend and forecast behavior (Abu-Akel and Shamay-Tsoory, 2011). A number of neuroimaging studies have demonstrated the crucial role of the MPFC in ToM tasks (Northoff and Bermpohl, 2004; Uddin et al., 2007; Qin and Northoff, 2011). Some researchers also declared that ToM was subserved by the posterior DMN (pDMN) regions. For instance, Saxe argued that the right TPJ was vital for representing mental states, particularly false beliefs (Saxe, 2006), and Samson and colleagues proposed that the left TPJ (coupled with the frontal lobes) was crucial for the representation of mental states (Samson et al., 2004). In general, neuroimaging studies have identified a common pattern of brain activation underlying autobiographical memory, ToM, and the DMN (Fair et al., 2008; Spreng et al., 2009; Spreng and Grady, 2010).

Past ToM studies investigating the brain connectivity within the DMN revealed strong connections between the parietal and frontal cortex. For instance, Atique and colleagues compared the different patterns of functional connectivity between inferring another person's emotion (emotion mentalizing) and intention (intention mentalizing) in the DMN. The results revealed a double dissociation, such that a more anterior region of the right and left TPJ was more strongly activated during emotion mentalizing and showed stronger FC with the vMPFC, whereas a more posterior region was more strongly activated during intention mentalizing (Atique et al., 2011). Burnett and Blakemore found that an anterior rostral region of the MPFC (arMPFC) showed greater connectivity with the posterior superior temporal sulcus (pSTS) bordering on the TPJ and anterior temporal cortex during social emotion (such as embarrassment and guilt) than basic emotion, which was in line with the assumption that social emotions require the representation of another's mental states. They also found that the adolescent group possessed stronger connectivity between arMPFC and pSTS/TPJ during social vs. basic emotion than did the adult group (Burnett and Blakemore, 2009). Moreover, Mason et al. (2008) detected that the autism group had lower functional connectivity within the DMN network (between the left medial frontal gyrus and right TPJ) in the intentional inference condition than the control group. In addition, researchers found that when subjects made the decision to punish in-group members and out-group members for violating social norms (third-party punishment), the less in-group members were punished, the stronger was the FC between the dMPFC and left TPJ (Baumgartner et al., 2012).

Some other studies explored the connectivity between the DMN network and other regions during ToM processing. For example, in a study asking schizophrenia patients to infer the social interactions of two moving triangles, FC analyses showed that the degree of FC between task-positive (lateral fronto-temporal network and insula) and task-negative (medial fronto-temporal network and pDMN) regions was significantly reduced in schizophrenia patients as compared to controls (Das et al., 2012). Another study also detected that autistic patients had lower FC between the DMN (the left MPFC and the right TPJ) and a left hemisphere language network (the inferior frontal gyrus and posterior left middle temporal gyrus) in the intentional inference condition than the control group (Mason et al., 2008). Additionally, in a study using an affective speech comprehension task, researchers identified three functional modules with FC analysis, including a “medial” ToM network (the MPFC and TPJ regions), a bilateral “language” network (the inferior frontal and temporal areas), and the bilateral amygdala. The cooperation of these modules was observed during people's emotional speech comprehension, with the left angular gyrus playing a critical role when the medial network and the language network interacted (Herve et al., 2012). Furthermore, Lombardo et al. (2010) found that the vMPFC, PCC/precuneus, and TPJ all exhibited the same FC patterns during mentalizing of both self and others, which indicated that identical neural circuits were implementing processes involved in the mentalizing of both self and others.

To sum up, the main findings of ToM studies focused on the connection between the dMPFC and TPJ (Mason et al., 2008; Burnett and Blakemore, 2009; Baumgartner et al., 2012; Herve et al., 2012), with few studies on the FC between vMPFC (Lombardo et al., 2010; Atique et al., 2011) and aMPFC (Burnett and Blakemore, 2009), as well as some other regions, such as the insula and language network (Das et al., 2012; Herve et al., 2012). Relative to emotion perception and empathy, ToM is considered as a relatively high-level cognitive process (Gallagher and Frith, 2003; Amodio and Frith, 2006). Many species can predict the goals of others, while only humans and perhaps some non-human primates can separate one's own mental perspective from that of others (Van Overwalle, 2009; Van Overwalle and Baetens, 2009). The process of ToM critically involves self-projection, since we must imagine ourselves in the same situation as another person and use our own experiences to simulate and understand the mind of that person (Blakemore and Decety, 2001; Spreng et al., 2009; Spreng and Grady, 2010; Spreng and Mar, 2012). Hence, the ToM processes require not only representing current and mnemonic event materials, which mainly depends on the posterior hemisphere of the human brain, but also distinguishing self from others, which is the critical function of the frontal cortex. The involvement of dMPFC in ToM is perhaps due to its responsibility for evaluation and decision-making processes in self- and other-referential processing (van der Meer et al., 2010).

### Morality

Psychologists' interest in the moral dimensions of life and thoughts could date back to the dialogs of Plato and Aristotle's ethical treatises. In the recent 20 years, neuroscience has started a new era for moral psychology. Neuroimaging studies have found several brain regions related to morality, such as the ACC (Greene et al., 2004), TPJ (Young et al., 2007, 2011; Young and Saxe, 2008, 2009), vMPFC (Tangney et al., 2007; Zahn et al., 2009; Moll et al., 2011), and dorsolateral prefrontal cortex Greene et al., 2004, 2008. The distributed nature of the moral network led researchers to shift their focus from seeking domain-specific brain regions dedicated to morality to determining the contributions of domain-general processes to morality (Shenhav and Greene, 2010; Young and Dungan, 2011). The existing results show that the moral brain network is closely associated with the DMN (Buckner et al., 2008; Bzdok et al., 2012; Reniers et al., 2012).

Connectivities within the DMN have been found in some morality studies. Decety found that the adult group showed the strongest connectivity between the vMPFC and pSTS/TPJ during viewing of moral actions relative to non-moral actions when compared to other, younger groups (Decety et al., 2012a). Harrison et al. (2008a) compared the FC within the DMN when subjects were resting, judging moral dilemmas, or performing the Stroop task. They found that regions within the DMN, particularly the posterior and anterior cingulated cortex, showed greater correlated activity during the moral dilemma task compared to the resting state. Pujol and colleagues further discovered that, in contrast with control subjects, psychopathic individuals with documented histories of severe criminal offenses showed significantly reduced FC between the medial frontal cortex (aDMN) and posterior brain areas (pDMN) in the resting state (Pujol et al., 2012).

Due to the complexity of morality, researchers are also very interested in the relation between the DMN and other networks, particularly the amygdala. When categorizing illegal and legal behaviors in an implicit association moral judgment task, youths with psychopathic traits displayed reduced FC between the amygdala and the medial OFC compared with healthy controls (Marsh et al., 2011). Decety et al. (2012a) found a positive age-related increase of FC between the vMPFC and amygdala in response to intentional harm. Another study reported significantly reduced fractional anisotropy (FA), an indirect measure of microstructural integrity, in the uncinate fasciculus (white matter connections linking the amygdala and OFC) of psychopaths compared with controls (Craig et al., 2009). Cocaine-dependent subjects have been found to have less resting-state functional connectivity between the ACC, thalamus, insula and the brain stem compared with controls (Verdejo-Garcia et al., 2012). Furthermore, researchers also found that the strength of the coupling between the dorsolateral premotor cortex and the DMN was positively correlated with the impulsivity scores in juvenile offenders but negatively correlated with age in typically developing individuals (Shannon et al., 2011).

Moral judgment is one of the most complex social behaviors. It involves a variety of lower level cognitive processes, such as distinguishing between self and others, integrating social norms, computing goal-directed actions, showing empathy to others and inferring the intentions of others (Moll et al., 2008; Bzdok et al., 2012; Feldmanhall et al., 2013). Corresponding complexity has been shown in the above FC results. Moral judgment studies reported FC results that not only involved areas subserving emotion perception, empathy, ToM, but also other regions, such as the FC between the medial OFC and precentral sulcus (Decety et al., 2008), as well as the ACC and thalamus (Verdejo-Garcia et al., 2012). However, neuroimaging studies using brain connectivity methods are still scarce in the field of morality. Given the importance of moral judgment to society, high priority should be given to conducting more studies using the FC approach to further explore the neural mechanisms of morality.

## Discussion

One of the consistent trends revealed in the above studies is that tasks from all the related fields of social understanding of others, from emotion perception to morality, elicit brain connectivity changes from the MPFC (extending to the ACC), a core region of the DMN, to other regions either inside (e.g., TPJ or PCC) or outside (e.g., insula or amygdala) of the DMN. Furthermore, more complex behaviors are subserved by brain regions which are situated higher in the frontal cortex. These results indicate that the MPFC plays a critical role in the social understanding of others, and different parts of MPFC take charge in distinct cognitive processes. According to Andrews-Hanna et al. (2010b), the MPFC can be divided into three subregions that belong to different subsystems of the DMN: the dMPFC in the dMPFC subsystem, the vMPFC in the MTL subsystem and the aMPFC in the midline common core system. The FC results reviewed in the current article provide support for the statements above.

### Connectivity from the vMPFC of the MTL subsystem

The vMPFC in the MTL subsystem is crucial in processing emotional features during social cognition. Connectivity changes between the vMPFC and other DMN regions (TPJ) have been found in ToM studies and morality studies. Atique and colleagues found that a more anterior region of the right and left TPJ showed strong FC with the vMPFC during emotion mentalizing (Atique et al., 2011). In contrast, Decety et al. (2012a) found an increase of FC between the vMPFC and pSTS/TPJ while viewing moral actions in adults compared to adolescents. The connection between the vMPFC and TPJ in these two fields can be attributed to the affective aspects of ToM that enables humans to infer emotions.

The dense connections between the vMPFC and emotional regions (e.g., amygdala, insula) means this frontal region can represent and regulate socioemotional states and synthesize a diverse range of information to represent affective mental states (Abu-Akel and Shamay-Tsoory, 2011). In all four fields, particularly emotion perception and empathy, the connectivity changes between the amygdala and vMPFC were repeatedly attested. The detection of connectivity between these two regions, to a certain extent, is consistent with discoveries in animal studies using fear conditioning paradigms which affirm that these regions play a critical role in the process of animal fear conditioning (Maren and Quirk, 2004; Jovanovic and Ressler, 2010; Fiorenza et al., 2012). Researchers have put forward a fear conditioning neuromechanism model, in which learning the conditioned responses in the central nucleus of the amygdala is modulated by two separate processes. One signals a positive prediction error from the basolateral amygdala, and another signals a negative prediction error from the vMPFC (Moustafa et al., 2013). This model is, in part, similar to the Integrated Emotion Systems (IES) model proposed by Blair (2007), which states that relatively independent emotion learning systems (e.g., the processing of fearful, sad and happy expressions in the amygdala, disgust expressions in the insula, as well as angry expressions in the inferior frontal cortex) input reinforcement expectancy information to the vMPFC while processing reinforcement provided by specific reinforcers of emotional expressions. The vMPFC represents the information and thus allows decision making, including moral decision making. The reduced connectivity between the MPFC and amygdala (Marsh et al., 2008; Glenn, 2011; Motzkin et al., 2011) instead of the insula and inferior frontal cortex in psychopaths relative to controls offers strong confirmation, as their impairments when processing care-based transgressions is thought to depend on the amygdala's role in the association of the transgression with the fear/sadness of the victim. Compared with the IES model, the amygdala-hippocampal-prefrontal interaction model includes the hippocampus, which is also frequently found in emotion related studies using functional connectivity methods (Kensinger and Corkin, 2004; Smith et al., 2006), and takes the effects of environment into account. However, there are still many open questions. For example, what is the actual role of the vMPFC? Does this region only signal a negative prediction error to the central nucleus of the amygdala, as Moustafa states, or does it play a part in successful decision making, as Blair asserts? How do other's emotions influence one's own moral decision?

### Connectivity from the aMPFC of the cortical midline structures

The aMPFC and PCC are part of the core cortical midline structures (CMS) of the DMN, which mostly contributes to the elaboration of the experiential feelings of self (Northoff et al., 2006, 2011; Leech et al., 2011; Pearson et al., 2011; Qin and Northoff, 2011; Denny et al., 2012; Leech and Sharp, 2013). The aMPFC has been proposed to be critical in making self-other distinctions. For example, the aMPFC activates when participants make judgments or remember trait adjectives about themselves compared to other people (e.g., Kelley et al., 2002; D'Argembeau et al., 2005; Heatherton et al., 2006; Mitchell et al., 2006). The above results show the crucial role the aMPFC plays in processes of social behavior, especially empathy. For instance, medical experts who applied painful procedures in their practice showed negative FC between the MPFC and AI, while controls showed no significant correlation with the MPFC (Cheng et al., 2007). It could be interpreted that long-term practice allows the medical experts to regulate the unpleasant feelings through self-other discrimination to identify the real protagonist of pain. In addition, observing a friend experience social exclusion triggers greater intensity of FC between the MPFC and both the dorsal ACC and bilateral insula than observing a stranger doing so (Meyer et al., 2013). This result can be explained by the logic that the concept of friend, as compared to stranger, is closer to the self, thus social exclusion of a friend brings about greater FC.

### Connectivity from the dMPFC of the dMPFC subsystem

The main results of the reviewed studies with regards to the DMN are the associations between the dMPFC and TPJ in the dMPFC subsystem, which were present not only in ToM (mentalizing) but also in morality studies. Understanding complex social interactions among people who are presumed to be social, interactive, and emotive always involves the processing of self-reflective thoughts and judgments (Buckner et al., 2008). Thus it is not surprising that connections between the TPJ and dMPFC are commonly found in these studies, since these two areas are key regions known to be involved in inferring temporary goals, intentions, desires, and more enduring dispositions of others owing to previous localization results using the mentalizing paradigm (Gallagher and Frith, 2003; Mitchell et al., 2005; Hampton et al., 2008; Steinbeis and Koelsch, 2009; Van Overwalle and Baetens, 2009). For example, studies have shown that functional connectivity between the dMPFC and TPJ increased when healthy participants performed ToM tasks on social properties but decreased when autistic participants did (Mason et al., 2008; Burnett and Blakemore, 2009; Baumgartner et al., 2012).

Several different theories have been proposed to interpret the relationship between the dMPFC and TPJ (as well as other LTC regions such as pSTS). For example, it is suggested that the dMPFC is associated with the internally-focused process of considering the contents of another person's mind, whereas those temporal regions are related to externally-focused processes that do not require consideration of a target's internal states (Lieberman, 2007). Some researchers have argued that the TPJ is responsible for a domain-general computational mechanism for reorienting attention to the agency (e.g., other individual) and the MPFC is more domain specific for understanding human mental states (Decety and Lamm, 2007). Others have proposed that the TPJ is more specific for the, possibly uniquely, human ability to reason about others' affective and cognitive mental states, and the MPFC is more domain-general (Saxe, 2006). Thus the FC between the TPJ and the MPFC would be an index of either shifting between internally-focused and externally-focused processes or communication between domain-general and domain-specific processing during the understanding of others' mental states.

### Connectivity from other regions of the DMN

Besides the MPFC and TPJ, several studies also revealed connectivity changes between the PCC/Precuneus in the CMS and other regions within and outside the DMN (Zaki et al., 2007; Harrison et al., 2008b; Assaf et al., 2010; Weng et al., 2010; Pujol et al., 2012). The PCC appears sensitive not only to explicit emotional engagement, for example, during tasks of emotional word processing and face-perception, but also implicit emotional engagement during self-directed attention or evaluation, as well as autobiographical memory Leech et al., 2011, 2012; Pearson et al., 2011; Leech and Sharp, 2013. Vogt et al. (2006) thus proposed that the PCC may respond to the general emotional content of events, particularly when the nature of processing is self-relevant.

In summary, during tasks from all four social fields, emotion perception, empathy, ToM, and moral judgments, connectivity changes were found between the MPFC and other regions within the DMN (e.g., TPJ, PCC) or outside the DMN (e.g., amygdala, insula). Evidence has shown that the MPFC is closely related to self-referential processing (Northoff et al., 2011; Wagner et al., 2012; Moran et al., 2013). The connectivity changes between the MPFC and other regions further confirm the viewpoint that humans use memories and associations from past experiences as the building blocks for understanding other's emotional and cognitive states. Furthermore, these studies suggest that different parts of the MPFC undertake distinct responsibilities. Specifically, connectivity changes between the emotion regions and vMPFC were repeatedly found in all four fields, particularly emotion perception and empathy; the aMPFC was found to be crucial, especially for empathy; and the associations between the dMPFC and TPJ were usually present in ToM (mentalizing) and morality studies. As social behaviors become more and more complex, the involvement of related regions in the medial frontal cortex gradually increased as well, which may reflect the transition of information processing from automatic to effortful cognitive processes. In consideration of all these findings, we propose that the vMPFC is engaged in identifying self-relevant information and assessing the salience of stimuli (Gusnard et al., 2001; Northoff and Bermpohl, 2004; Northoff et al., 2006); the aMPFC takes charge in making clear self-other distinctions (Andrews-Hanna et al., 2010b), and the dMPFC is involved in the evaluation and decision of whether a certain stimulus is applicable to the self or to another (van der Meer et al., 2010).

In addition to the MPFC regions, social understanding of others also includes cognitive processing for extracting existing storage and perceiving immediate material to represent current events, as well as for identifying and expressing the emotion itself. The former is closely related to the TPJ, which is believed to help in the establishment of a social context for a decision (Carter and Huettel, 2013), whereas the latter is managed by the amygdala, insula and other emotion regions. These three basic processes interact with each other and eventually lead to the formation of complex social behavior.

Reproducibility is a lingering issue with previous studies. For example, Andrews-Hanna and colleagues divided the MPFC into dMPFC, vMPFC, and aMPFC and proposed that they respectively belong to the dMPFC subsystem, MTL subsystem, and common core system (Andrews-Hanna et al., 2010a,b). van der Meer and colleagues further suggested that “the vMPFC is responsible for tagging information relevant for “self,” whereas the dMPFC is responsible for evaluation and decision-making processes in self- and other-referential processing” (van der Meer et al., 2010). However, other studies did not emphasize the role of aMPFC, but instead showed that the vMPFC responds more to self, whereas the dMPFC responds more to others (Denny et al., 2012; Wagner et al., 2012). Compared with these studies, the present article specifically highlights the function of self-other distinction in the aMPFC for two main reasons: theoretically, there must be some transition from self to others and the aMPFC anatomically connects the vMPFC and dMPFC; in practice, as we have presented, this area has been repeatedly found to participate in the differentiating of self and others. However, to address the divergence and inconsistencies between studies, more brain connectivity methods such as those from graph theory, statistical physics, and non-linear dynamics should be put to use to confirm the relations and differences between the subregions of the MPFC and the DMN. Transcranial magnetic stimulation and transcranial direct-current stimulation should also be considered because they can provide causal evidence to evaluate the above theories.

## Conclusion and future directions

In this article, we reviewed recent studies on the social understanding of others using brain connectivity methods. We focused on the brain connectivity within and outside the DMN in four different research fields: emotion perception, empathy, ToM, and morality. The reviewed studies suggest that the MPFC plays a key role in the social understanding of others, the subregions of the MPFC contribute differently to this function according to their roles in the different subsystems of the DMN, and more complex behaviors are related to anatomically higher regions in the frontal cortex. Starting from the bottom, the vMPFC in the MTL subsystem and its connection with emotion regions are mainly associated with emotion engagement during social interactions. Above the vMPFC, the aMPFC in the CMS and its connections with the PCC and ACC contribute mostly to making self-other distinctions. At the top, the dMPFC in the dMPFC subsystem and its connection with the TPJ are primarily associated with understanding others' mental states. Besides the MPFC and TPJ, the connectivities of the PCC also show some changes during tasks from the four social fields. These results indicate that the DMN is indispensable in the social understanding of others.

Several points require attention during future development of large-scale brain connectivity studies of social cognition. First of all, interest in brain connectivity arose from the study of brain lesions and neuropsychiatric disorders ranging from epilepsy to autism (Menon, 2011; Shafi et al., 2012). A rich body of literature on neuropsychiatric disorders suggest that abnormalities in the interactions of network components play a vital role in these disorders (Lytton, 2008; Vissers et al., 2012), and damage to specific functional connectivity networks can result in corresponding neuropsychopathy (Seeley et al., 2009). However, compared with lesions and patient studies, there are far fewer studies on healthy human participants applying the methods and theories of brain connectivity, let alone in the field of social cognition. This is a very promising approach for future work.

Secondly, most previous studies exploring the social brain in healthy participants only computed the functional or effective connectivity among regions of interest determined by prior experience or localization tasks, whereas a wide range of brain connectivity methods such as those from graph theory, statistical physics, and non-linear dynamics have been adopted in neuropsychiatric disorders studies (van den Heuvel and Hulshoff Pol, 2010; Menon, 2011; Xia and He, 2011; Stam and van Straaten, 2012; Yu et al., 2012; Griffa et al., 2013). Undoubtedly, these methods should be put to use to confirm the relations and differences between subregions in the MPFC or the DMN and deeply explore the complex social brain network in healthy participants.

Thirdly, so far most brain connectivity studies are conducted with fMRI, a technique based mainly on correlational evidence. However, investigating causality is the main goal of scientific studies, so building causal models accounting for the entire loop of social information processing within and between brains would be a promising future direction (Singer, 2012). Consequently, the methods for studying brain networks could be combined with many other methodologies, such as multi-voxel pattern analyses (Carter et al., 2012), transcranial magnetic stimulation/transcranial direct-current stimulation (Young et al., 2010; Carter et al., 2012), genetic-imaging approaches (Glenn, 2011), and pharmacological interventions (Sripada et al., 2012) to explore the neural substrates of various human physiological and psychological states during social interaction.

### Conflict of interest statement

The authors declare that the research was conducted in the absence of any commercial or financial relationships that could be construed as a potential conflict of interest.
